# Differences in the characteristics and functions of brain and spinal cord regulatory T cells

**DOI:** 10.1186/s12974-024-03144-1

**Published:** 2024-06-01

**Authors:** Mahiro Watanabe, Ako Matsui, Natsumi Awata, Ayame Nagafuchi, Mio Kawazoe, Yoshihiro Harada, Minako Ito

**Affiliations:** Division of Allergy and Immunology, Medical Institute of Bioregulation, Kyushu University, 3-1-1 Maidashi, Higashi-Ku, Fukuoka, 812-8582 Japan

**Keywords:** Tissue Tregs, EAE, Brain, Spinal cord

## Abstract

T cells play an important role in the acquired immune response, with regulatory T cells (Tregs) serving as key players in immune tolerance. Tregs are found in nonlymphoid and damaged tissues and are referred to as “tissue Tregs”. They have tissue-specific characteristics and contribute to immunomodulation, homeostasis, and tissue repair through interactions with tissue cells. However, important determinants of Treg tissue specificity, such as antigen specificity, tissue environment, and pathology, remain unclear. In this study, we analyzed Tregs in the central nervous system of mice with ischemic stroke and experimental autoimmune encephalomyelitis (EAE), a mouse model of multiple sclerosis. The gene expression pattern of brain Tregs in the EAE model was more similar to that of ischemic stroke Tregs in the brain than to that of spinal cord Tregs. In addition, most T-cell receptors (TCRs) with high clonality were present in both the brain and spinal cord. Furthermore, Gata3^+^ and Rorc^+^ Tregs expressed TCRs recognizing MOG in the spinal cord, suggesting a tissue environment conducive to *Rorc* expression. Tissue-specific chemokine/chemokine receptor interactions in the spinal cord and brain influenced Treg localization. Finally, spinal cord- or brain-derived Tregs had greater anti-inflammatory capacities in EAE mice, respectively. Taken together, these findings suggest that the tissue environment, rather than pathogenesis or antigen specificity, is the primary determinant of the tissue-specific properties of Tregs. These findings may contribute to the development of novel therapies to suppress inflammation through tissue-specific Treg regulation.

**Supplementary Information:**

The online version contains supplementary material available at 10.1186/s12974-024-03144-1.

## Introduction

Regulatory T cells (Tregs) are crucial for maintaining peripheral tolerance and tissue homeostasis in the immune system. These cells express the master transcription factor FoxP3 and suppress excessive immune responses by recognizing a variety of self-antigens and foreign antigens [1, 2]. Tregs in various nonlymphoid tissues, such as fat, skin, lung, intestine, heart, and brain, are termed “tissue Tregs” and play essential roles in tissue homeostasis and repair via tissue-cell interactions [3, 4]. They share common features, such as the IL-33 receptor ST2, the growth factor amphiregulin (Areg), and the anti-inflammatory cytokine IL-10 but also possess tissue-specific features, such as chemokine receptors, notch receptors, and neuropeptides [4]. After accumulation in damaged tissues such as muscle, fat, and the brain through specific antigen recognition [5, 6], their phenotype is thought to be influenced by the local microenvironment. Single-cell RNA sequencing technology has shown that tissue Tregs gradually acquire their characteristics in the corresponding lymph nodes and mature within the tissue [7–9].

In the context of central nervous system (CNS), brain Tregs, similar to those from other tissues, are Helios^+^ thymic-derived Tregs (tTregs) with a specialized TCR repertoire and high expression of CTLA-4, PD-1, Areg, and ST2—molecules associated with immune suppression, tissue repair, and anti-inflammatory responses. Our research shows that Tregs accumulate in the brain during the chronic period two weeks after stroke onset and are important for the recovery of neurological symptoms [5]. Increasing the number of Treg cells by overexpressing IL-2 in the IL-2:IL-2 antibody complex or astrocytes after stroke restores neurological function in the long term [10]. These reports suggest that Tregs may be potential therapeutic targets for neurological recovery after stroke.

Multiple sclerosis (MS) is an autoimmune disease in which autoreactive immune cells recognize and attack the protective myelin sheath surrounding nerves in the CNS. This causes irreversible damage that can impair movement, muscle control, vision, and cognition. Experimental autoimmune encephalomyelitis (EAE) is a representative model of MS characterized by paralysis resulting from CNS inflammation, neuronal demyelination, axonal damage, and neurodegeneration. Tregs produce IL-10 and TGFβ, which are important for EAE recovery [11] and support myelin regeneration. They directly signal glial precursors to differentiate into myelin-producing glial cells, which is a critical step in the remyelination process [12]. Tregs from patients with MS show defects in their suppressive capacity, including decreased expression of several functionally important genes, such as CTLA-4 and FoxP3, which may be responsible for disease development.

Thus, research on different tissue-specific Tregs has progressed, and their characteristics have become clear. However, the mechanisms by which each tissue Tregs acquire these specific properties have not been determined, and the major determinants of Treg tissue specificity, such as antigen specificity, tissue environment, and pathology, have not yet been determined. In this study, we aimed to elucidate the mechanism by which Tregs in the CNS acquire tissue-specific properties by employing a mouse model of ischemic stroke and EAE.

## Methods

### Mice

Wild-type C57BL/6J mice were purchased from KBT Oriental (Breeder: Jackson Laboratory Japan). Foxp3^hCD2KI^ mice, IL-17a-GFP mice and DEREG mice were generated as described previously [26, 27]. All mice were housed in a specific pathogen-free animal facility at Kyushu University with a 12-h light cycle. All the experimental procedures were reviewed and approved by the Kyushu University Animal Experiment Committee, and the care of the animals was in accordance with institutional guidelines.

### Mouse model of ischemic stroke

Male mice aged 8–12 weeks and weighing 20–30 g were used for focal brain ischemia experiments [5]. There was no significant difference in weight or age between the wild-type mice and any of the knockout groups. We used a transient MCAO model constructed using an intraluminal suture. This transient suture MCAO model was established as previously described [5]. The mice were anesthetized with isoflurane in a mixture of 70% nitrous oxide and 30% oxygen. During the MCAO procedure, the head temperature was maintained at 36 °C using a heat lamp. Sixty minutes after MCAO, the brain was reperfused by removing the intraluminal suture. Neurological function was evaluated using a previously described four-point scale neurological score method (0, no observable deficit; 1, forelimb flexion; 2, decreased resistance to lateral push without circling; 3, same behavior as grade 2, with circling) [28].

### Mouse model of EAE

#### Immunization of MOG_33-55_ peptides

Emulsions were prepared by mixing 200 μg of myelin oligodendrocyte glycoprotein peptide (MOG_33-55_) with complete Freund's adjuvant plus *M. tuberculosis*. For each mouse, 200 μg of the emulsion was injected subcutaneously into 3 sites on the back of Foxp3^hCD2KI^ and WT mice (2–3 months old, female). Pertussis toxins (500 ng/body) were injected intraperitoneally twice, on days 0 and 2.

#### Clinical score scoring method

0: normal, 0.5: decreased tail tonus, 1: complete tail droop, 1.5: hind limb weakness, 2: incomplete hind limb paralysis, 3: complete hind limb paralysis, 4: complete hind limb paralysis including incomplete forelimb paralysis, 5: death.

### Isolation of cells from the adult mouse brain and spinal cord

Mice were perfused with PBS transcardially, and the cerebrum, cerebellum and spinal cord were removed. For isolation of CNS Treg cells, the cerebrum, cerebellum and spinal cord were minced and enzymatically dissociated with 2 mg/ml collagenase D (Roche Applied Science) and 1 mg/ml DNase I in RPMI-1640 for 30 min at 37 °C. The digested tissue was centrifuged and resuspended in 37% Percoll (Cytiva) and centrifuged at 800 × *g* for 20 min, after which the cell pellets were collected. The isolated cells were stained with a fluorochrome-conjugated antibody. Fluorescein isothiocyanate (FITC), phycoerythrin (PE), peridinin chlorophyll protein-cyanine 5.5 (PerCP-Cy5.5), allophycocyanin (APC), PE-Cy7, and APC-Cy7-conjugated antibodies were purchased from BioLegend, BD or eBioscience. The primary antibodies used were as follows: anti-mouse CD45.2 antibody, anti-mouse CD11b antibody, anti-mouse CD3ε antibody, anti-human CD2 antibody, anti-mouse CD4 antibody, anti-mouse Areg antibody (R&D; BAF989), anti-mouse ST2 antibody, and anti-mouse HTR7 antibody (Novus; 56,352), anti-mouse Rorgt, anti-mouse Gata3, anti-mouse IL-1R, anti-mouse CD25, anti-mouse Lag3, anti-mouse CXCR6, anti-mouse CCR8, anti-mouse CXCR4, anti-mouse CD304 (Neuropilin1), and anti-mouse Helios. The fixable viability dye eFluor 780 (FVD780) was used to remove dead cells. The stained cells were sorted by FACSMelody, LSRFortessa (BD Biosciences) and FlowJo software (Tree Star). The gating strategy for Tregs was FVD^−^CD45.2^+^CD11b^−^CD3^+^CD4^+^Foxp3^+^. For Areg staining, cells were stained after stimulation with eBioscience™ Cell Stimulation Cocktail (Thermo Fisher Scientific) for 3 h.

### Immunohistochemistry

Mice were anesthetized by inhalation with isoflurane and perfusion was performed by injecting PBS into the left ventricle. The lumber spinal cord segments were dissected and immersed in 4% paraformaldehyde (PFA) and fixed at 4 °C for 24 h. For paraffin-embedded sections, the spinal cords in cassettes were prepared by the Laboratory for Research Support, Medical Institute of Bioregulation, Kyushu University. Samples were embedded in paraffin and cut into 5 µm sections. Samples on slides were de-waxed in xylol and ethanol, antigen retrieval was performed using boiled citrate buffer and samples were blocked for 1 h at room temperature (25 °C) using Blocking One Histo (nacalai tesque). The sections were incubated with primary antibodies included in 5% BSA/PBS overnight at 4 °C: rabbit anti-MBP (1:300; Cell Signaling Technologies, 78,896), mouse anti-GFAP (1:500; Cell Signaling Technologies, 3670), goat anti-Iba1 (1:300; FUJIFILM Wako Pure Chemical Corporation, 011–27991). After primary antibody incubation, sections were washed twice with PBS containing 0.1% Triton X-100 (PBS-T) and once with PBS, and incubated with the secondary antibody and Hoechst for 30 min at room temperature: donkey anti-rabbit Alexa Fluor 647, donkey anti-mouse Alexa Fluor 546, donkey-goat Alexa Fluor 488 (all 1:300; Invitrogen), then washed with PBS-T and PBS. Sections were sealed with cover glasses using PermaFluor mounting medium (Thermo Fisher, TM-030-FM). The images were taken with BZ-X700 (Keyence) and image analysis was performed using ImageJ. Statistical analysis was performed in Prism9 (Graphpad).

### In vivo depletion of Treg cells in EAE mice

DEREG or wild-type littermates were injected with diphtheria toxin (Merck) intraperitoneally at 20 μg/kg on days 13 and 15 after immunization with MOG. At day 17, these mice were sacrificed, and brains and spinal cords were analyzed by flow cytometry.

### Transferring Tregs into mice with EAE

EAE was induced in Foxp3^hCD2KI^ mice, and Tregs were sorted from the brain and spinal cord of EAE mice at day 23 after immunization. As a control, Tregs from the lymph nodes of untreated mice were sorted. These Tregs (2 × 10^4^ cells) from the brain or spinal cord of 40 Foxp3^hCD2KI^ mice were injected intravenously into WT recipients 5 days after immunization, and neurological symptoms were observed at the appropriate time points. (Recipients of LN Treg: N = 14, EAE brain Treg: N = 14, EAE SC Treg: N = 16).

For transfer experiment of Tregs after onset of EAE (Fig. S8), EAE was induced in Foxp3^hCD2KI^ mice, and Tregs were sorted from the brain and spinal cord of EAE mice at day 23 after immunization. As control, Tregs from the spleens of 4 CFA-treated mice were sorted. These Tregs (2 × 10^4^ cells) from the brain or spinal cord of 34 Foxp3^hCD2KI^ mice were injected intravenously into WT recipients 17 days after immunization, and neurological symptoms were observed at the appropriate time points. (Recipients of PBS: N = 6, CFA-treated SP Treg: N = 6, EAE brain Treg: N = 7, EAE SC Treg: N = 6).

### Chemokine array

EAE was induced in 2–3-month-old C57BL/6 J mice. On day 23, after the mice were euthanized and perfused with PBS, the cerebrums and spinal cords were extracted and crushed by a bead shocker for 20 s at 5,000 rpm three times. The samples were centrifuged for 10 min at 15,000 rpm and 4 °C, after which the supernatants were collected. A chemokine array was performed using the Proteome Profiler Mouse Chemokine Array Kit (R&D; ARY020) according to the manufacturer’s instructions. The chemiluminescence signals on the membranes were detected by ChemiDoc (Bio-Rad). The data analysis was performed with Image Lab (Bio-Rad).

### Migration assay

A migration assay was performed using a CytoSelect™ 96-well cell of migration assay kit (Cell Biolabs; CBA-105) according to the manufacturer’s instructions. Tregs isolated from the brains or spinal cords of EAE mice were seeded at 2 × 10^4^ cells/well in 10% FBS medium supplemented with 0.2 mg/ml CCL6, CCL12, or CCL1 (BioLegend). At 4 h after seeding, the migrating cells were lysed with lysis buffer/CyQuant^®^ GR dye, and fluorescence was measured with an EnSpire^®^ Multimode Plate Reader (PerkinElmer).

### TCR cloning and IL-2 ELISA

TCR sequences were obtained from scTCR-seq information from brain or spinal cord Tregs and 2D2 or OT-II mice. The DNA was synthesized by eBlocks Gene Fragments (IDT). The α and β chains were linked by the P2A sequence and inserted into the pMX-IRES-GFP vector. The retroviruses generated by these pMX-TCR-IRES-GFP vectors were infected into TG40 cells. GFP-expressing cells were sorted by FACSMedoly and cocultured with bone marrow-derived dendritic cells in media supplemented with MOG_33-55_ peptide (5 μg/ml), OVA peptide (200 μg/ml) or anti-CD3ε/CD28 antibodies (2 μg/ml) for 24 h. IL-2 in the supernatants was detected by a mouse IL-2 ELISA kit (Invitrogen; 88–7024) according to the manufacturer’s instructions.

### RNA sequencing

#### Cell isolation and RNA extraction

Bulk RNA-seq of cerebrum, cerebellum and spinal cord tissues from EAE mice: EAE was induced in 2- to 3-month-old C57BL/6J mice. On day 18 and 32 post immunization, after the mice were euthanized, the cerebrum, cerebellum and spinal cord were extracted from 12 mice (day 18: pooled 3 mice × 2 samples, day 32: pooled 3 mice × 2 samples) and homogenized after PBS perfusion. The tissues were homogenized in PBS by beads shocker (TOMY, MS-100). RNA was extracted from a portion of the tissues using an RNeasy Mini Kit (QIAGEN).

Bulk RNA-seq of Tregs: Foxp3^hCD2KI^ mice were induced to experience ischemic stroke or EAE (stroke: male 4 mice × 2, EAE brain and spinal cord: female 4 mice × 2, spleen: 2 mice × 2). At day 28 after ischemic stroke onset or immunization, the mice were perfused with PBS, and the brains and spinal cords were homogenized. The immune cells were isolated using the methods described in “Isolation of cells from the adult mouse brain and spinal cord”. Treg sorting was performed using a FACSAriaIII, and RNA extraction was performed using an RNeasy Plus Micro Kit (QIAGEN).

#### RNA sequencing

Library preparation was performed using NEBNext PolyA stranded Ultra II Directional RNA Library Prep (New England BioLabs, MA) by Azenta. The libraries were sequenced on Illumina NovaSeq 6000 sequencers (Illumina, San Diego, CA). Sequence reads in FASTQ format were applied to FastQC (v0.11.4) to assess sequence quality. For adapter trimming, Trim Galore! (v0.6.0) was used. The sequence reads were mapped to the mouse reference genome GRCm38/mm10 from Ensembl (release 102) using HISAT2 (version 2.2.1). The mapped reads were counted for genes using featureCounts (v.2.0.0). The data analysis was performed by iDEP.96. Differentially expressed genes (DEGs) obtained between clusters were subjected to Gene Ontology (GO) analysis using Metascape (v3.5).

### Single-cell RNA-seq

#### Cell isolation

Two- to three-month-old C57BL/6 J mice were subjected to EAE or cerebral infarction. After the mice were euthanized, the cerebrum, cerebellum, spinal cord and lymph nodes were removed by PBS perfusion and homogenized. Staining was performed with the following antibodies: anti-CD45.2, anti-CD11b, anti-hCD2, anti-CD3ε, and anti-CD4. Totalseq hashtag antibody (BioLegend) was then reacted with each sample, and Tregs were sorted with a FACSMelody (BD Biosciences).

#### Library preparation and NGS

Libraries were prepared according to the protocol of the Chromuim Next GEM Single Cell 5' Reagent Kits v2 (Dual Index, 10 × Genomics, Pleasanton, CA, USA) using a Chromium controller (10 × Genomics). Briefly, GEMs were prepared by loading barcoded Single Cell VDJ 5' Gel Beads, master mix containing cells and RT enzymes, and Oil onto Chromium Next GEM Chip K. Barcoded cDNA was subsequently generated from the GEM. The cDNA was amplified via PCR to generate V(D)J, 5' Gene Expression and Feature Barcode libraries. Sequencing was then performed using a NovaSeq 6000 (Illumina, San Diego, CA, USA) at the Laboratory for Research Support of the Institute of Bioregulatory Medicine, Kyushu University.

#### Preprocessing

Fastq files were generated from the NGS data using Cell Ranger (v6.1.2) (10 × Genomics). The c.loupe files (gene expression) and v.loupe files (TCR sequence) were obtained from Cell Ranger Multi (mouse genome; Genome Reference Consortium Mouse Build 38, mm10).

#### 4Data analysis

The Loupe Browser (v7.0.1) and Loupe V(D)J Browser (v5.1.0) were used to analyze gene expression and the TCR repertoire. Differentially expressed genes (DEGs) obtained between clusters were subjected to Gene Ontology (GO) analysis using Metascape (v3.5).

### Statistics

All the experiments were performed with at least two biological replicates and yielded comparable results except for the scRNA-seq experiment, which was conducted only once. Sample sizes were determined based on previous similar experiments [5] but were not predetermined by any statistical methods. The data are expressed as the mean ± s.e.m. Statistical significance was determined by one-way or two-way analysis of variance (ANOVA) followed by post hoc Tukey’s multiple comparisons test to analyze differences among three or more groups and by unpaired Student’s t test to analyze differences between two groups. P < 0.05 was considered to indicate a significant difference (*p < 0.05; **p < 0.01; ***p < 0.001; ****p < 0.0001; ns, not significant). All the statistical analyses were performed with GraphPad Prism (version 9.5.1).

## Results

### Tregs prevent the worsening of neurological symptoms in EAE mice

**Fig. 1:**
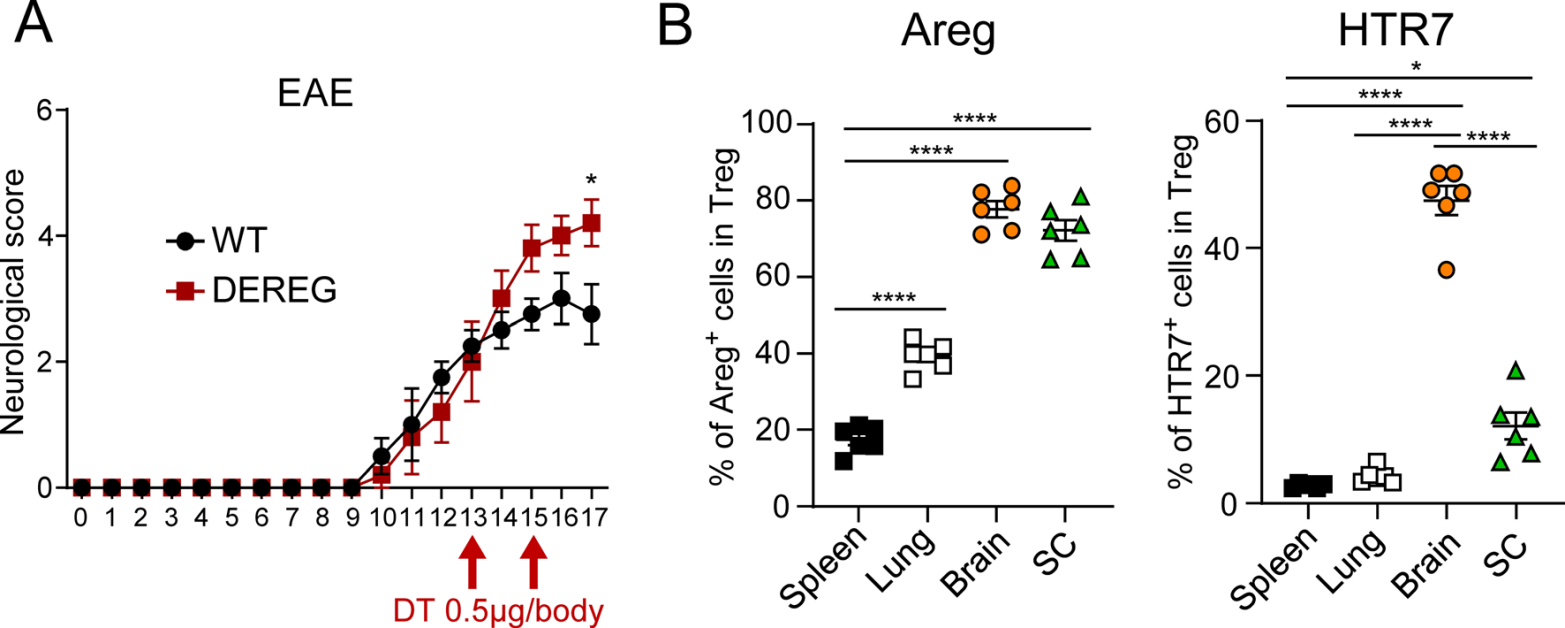
Effect of Tregs on the recovery of neurological symptoms in EAE mice. **A** Neurological score of EAE-induced WT (N = 3) or DEREG (N = 6) mice treated with diphtheria toxin at 13 and 15 days after immunization with MOG. **B** Flow cytometric analysis of Tregs in the brain, spinal cord of EAE model mice (N = 6) at day 24, lung and spleen of control mice (N = 6). The data are representative of at least two independent experiments. *P* values were determined by two-way ANOVA (**A**) and one-way ANOVA (**B**). The data are shown as the mean ± s.e.m. SC: spinal cord

We previously reported that Tregs contribute to the recovery of neurological symptoms after ischemic stroke [5]. To investigate whether Treg dysfunction or absence was also involved in pathologies other than ischemic stroke, we induced EAE in DEREG mice. These mice express the diphtheria toxin receptor under the control of the Foxp3 gene promoter/enhancer [13]. The depletion of Tregs by the administration of diphtheria toxin post onset resulted in the exacerbation of neurological symptoms (Fig. 1A, S1A), indicating the importance of Tregs in suppressing the progression of EAE.

The EAE model used in this study is initially induced through spinal cord inflammation, followed by subsequent damage to the brain. We examined the characteristics of Tregs in the brain and spinal cord of mice with EAE and found that the expression levels of Areg, known tissue Treg marker, were high in both the brain and spinal cord, whereas that of HTR7, which we identified as a brain Treg marker, was high only in the brain (Fig. 1B). However, the number of Areg^+^, HTR7^+^ Tregs was higher in the spleen due to the overwhelmingly higher number of Tregs in the spleen (Fig. S1B). These results indicate that Treg characteristics differ between the brain and the spinal cord, even in mice with the same pathology.

### Analysis of CNS Tregs in mice with stroke and EAE

**Fig. 2:**
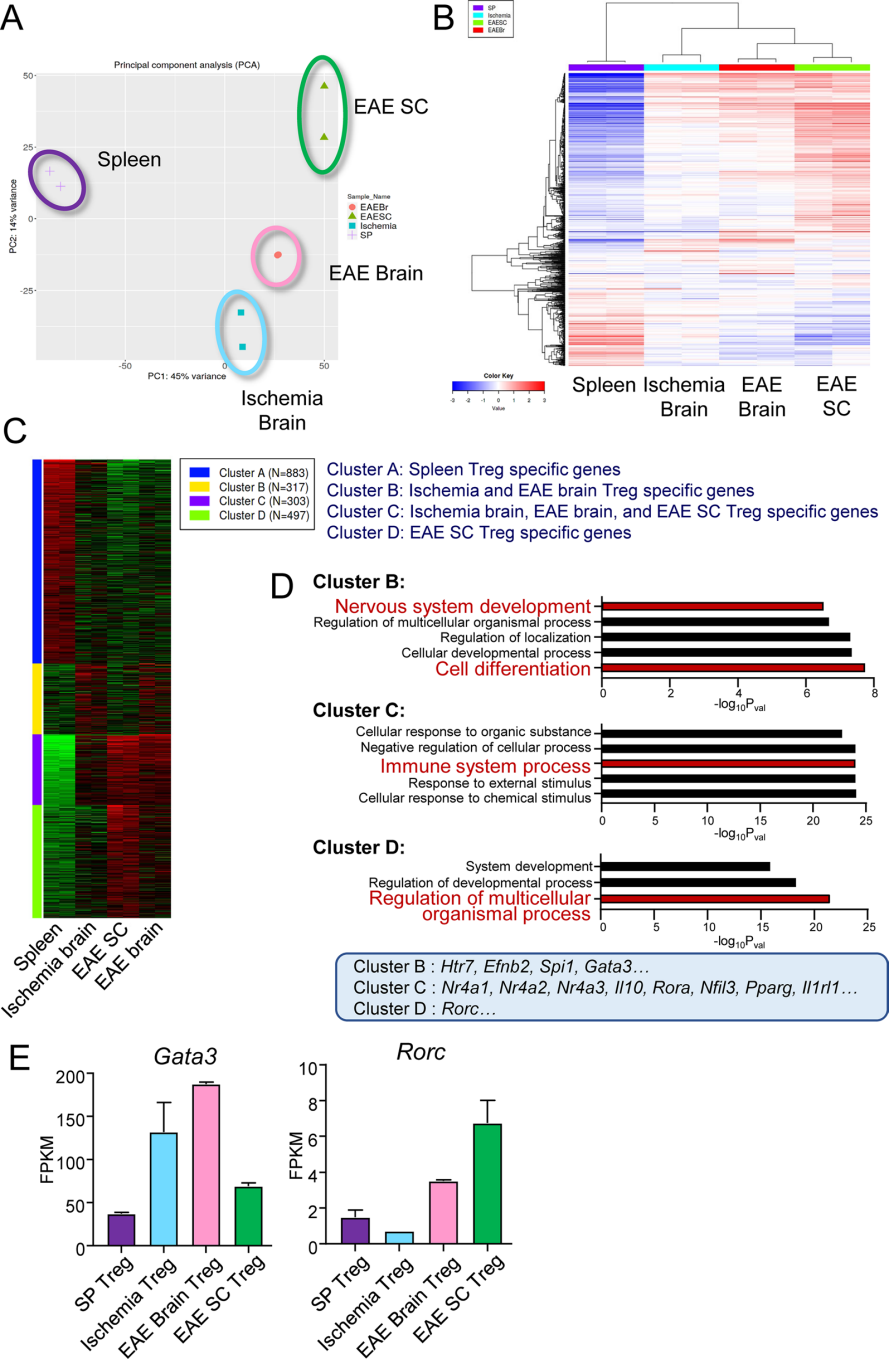
Characterization of Tregs in the brain and spinal cord by bulk RNA-seq. **A**, **B** PCA (**A**) and heatmap (**B**) of bulk RNA-seq analysis of Tregs from brain and spinal cord of ischemic or EAE Foxp3^hCD2KI^ mice at day 28 and Tregs from spleen of control Foxp3^hCD2KI^ mice (N = 2, pooled from 4 animals for each replicate). **C**, **D** Heatmap (**C**) and bar graph (**D**) of Gene Ontology (GO) analysis results for the DEGs in the four clusters and the top 5 GO terms. **E** The expression levels of *Gata3* and *Rorc* in Tregs determined from bulk RNA-seq data of the indicated tissue Tregs

Next, gene expression analysis of CNS Tregs from ischemic stroke and EAE mice (brain and spinal cord separately) was performed to determine their pathology- and tissue-specific characteristics. We sorted CNS Tregs and performed bulk RNA-seq analysis (Fig. S2A, B). Principal component analysis (PCA) of bulk RNA-seq data revealed that Tregs in the brain and spinal cord have different characteristics from those in the spleen, a secondary lymphoid tissue, and that EAE brain Tregs were more similar to brain Tregs in ischemic stroke than spinal cord Tregs in EAE (Fig. 2A). Enrichment analysis of genes characterizing PCA eigenvectors confirmed that genes related to the nervous system and cytokines are key variables (Fig. S2C). A heatmap revealed many genes that were specifically expressed in EAE spinal cord Tregs (Fig. 2B).

To clarify the differences between brain and spinal cord Tregs, we used Gene Ontology (GO) analysis to classify the genes into four clusters: Cluster A, specific for spleen Tregs; Cluster B, specific for brain Tregs in stroke and EAE; Cluster C, highly expressed in all CNS Tregs in stroke and EAE; and Cluster D, highly expressed in spinal cord Tregs only (Fig. 2C). Brain Treg-specific Cluster B exhibited increased expression of genes related to nervous system development (including *Htr7*) and cell differentiation (*Efnb2*, *Spi1*, and *Gata3*). Cluster C included genes characteristic of tissue Tregs, such as *Nr4a1*, *Nr4a2*, *Nr4a3*, *Il10*, *Rora*, *Nfil3*, *Pparg*, and *Il1rl1*. Cluster D showed an increased expression of genes such as *Rorc* and *Il17f* (Fig. 2D, S2D). The expression levels of *Gata3* and *Rorc* were considered representative of genes that differed between the brain and spinal cord (Fig. 2E). Thus, these results reveal distinct gene expression patterns between brain and spinal cord Tregs within the same CNS. This suggests that some Treg characteristics may be more similar in the same tissue in different pathologies than in different tissues in the same pathology.

**Fig. 3:**
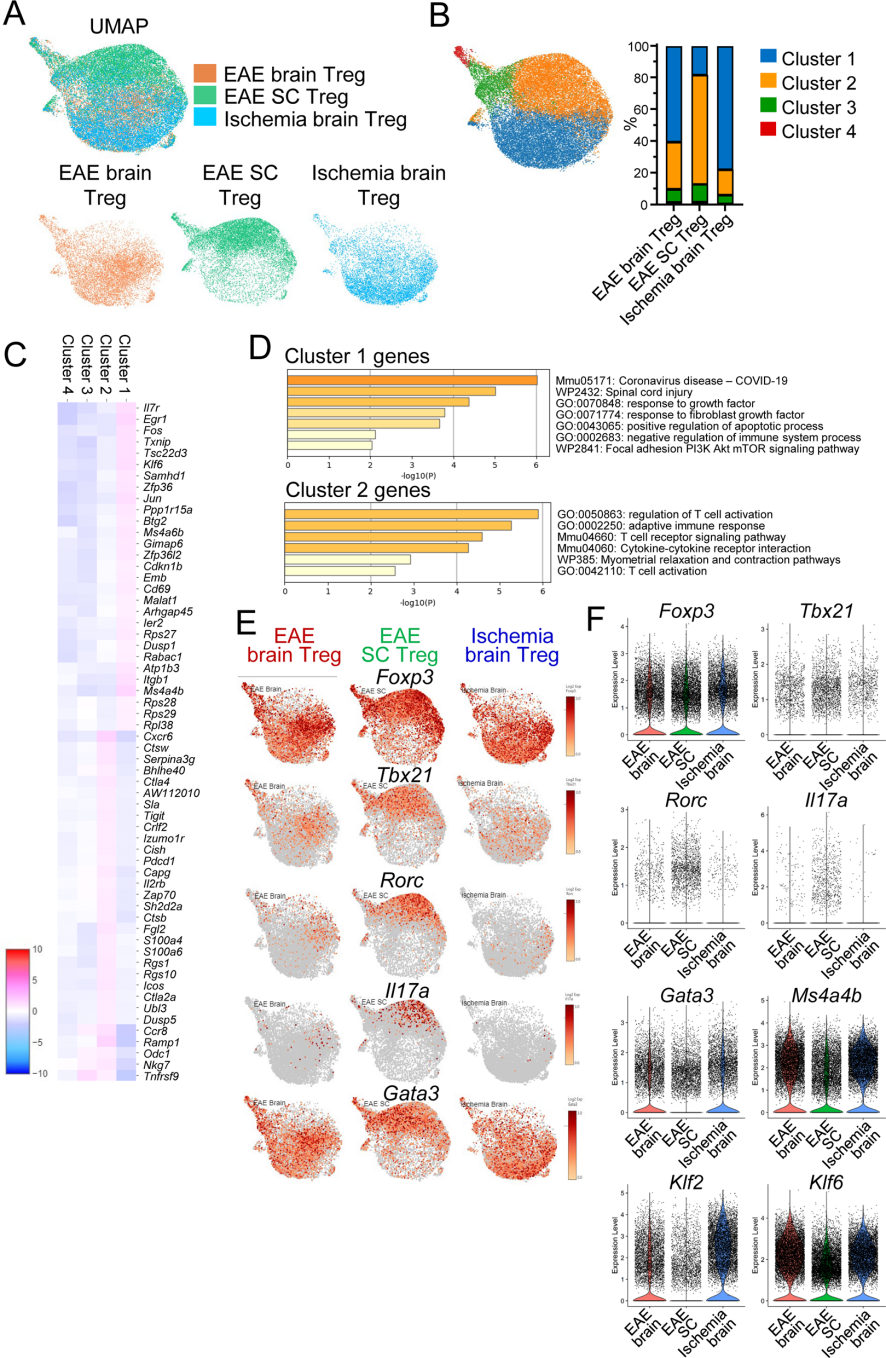
Characterization of Tregs in the brain and spinal cord by scRNA-seq. **A-F** scRNA-seq data of Tregs derived from EAE Foxp3^hCD2KI^ mice (N = 4) at day 23. Uniform manifold approximation and projection (UMAP) visualization of scRNA-seq data of Tregs divided by the ischemic brain, EAE brain, and EAE spinal cord (**A**). UMAP plot of scRNA-seq data for Tregs divided into 4 clusters and a bar plot of the percentage of clusters (**B**). Gene expression heatmap of the signature genes per cluster. This figure shows only cluster 1 and cluster 2 (**C**). GO analysis of DEGs in cluster 1 and cluster 2 (**D**). Feature plot (**E**) and violin plot (**F**) of the expression levels of several genes, including those of the transcription factors *Foxp3*, *Tbx21*, *Rorc*, and *Gata3*, in Tregs in the brain or spinal cord

Next, scRNA-seq was performed to eliminate the possibility of contamination from tissue resident cells during sorting and to compare gene expression at the cellular level. Uniform manifold approximation and projection (UMAP) showed that EAE brain Tregs had different characteristics from EAE spinal cord Tregs but were similar to stroke brain Tregs (Fig. 3A). When the Treg population was divided into four clusters, most EAE and ischemic brain Tregs were in Cluster 1, whereas most spinal cord Tregs were in Cluster 2 (Fig. 3B). A heatmap illustrating the comparison of differentially expressed genes between the clusters highlighted genes specific to brain or spinal cord Tregs (Fig. 3C). Enrichment analysis revealed that Cluster 1 had high expression of genes related to growth factors and negative regulation of the immune system, whereas Cluster 2 had high expression of genes related to T cell activation (Fig. 3D). Differences in Treg cell function mediated by transcription factors have been reported [14]. According to the analysis of the gene expression of transcription factors involved in T-cell subtype classification, *Foxp3*, a master regulatory gene of Tregs, was highly expressed across all groups and was expressed at similar levels in Tregs (Fig. 3E, F). Additionally, the expression of *Tbx21*, a regulator of the Th1 cytokine interferon gamma (IFN-γ), and *Rorc*, which is involved in *IL-17a* expression, was elevated in EAE spinal cord Tregs (Fig. 3E, F, S3A). On the other hand, brain Tregs presented increased expression of *Gata3*, a transcription factor important for tissue Tregs and Th2 cells, along with genes that antagonize effectors such as *Klf2* and *Klf6* [15] (Fig. 3E, F). These results suggest that brain and spinal cord Tregs have distinct characteristics.

### TCR analysis of the brain and spinal cord of EAE mice

**Fig. 4:**
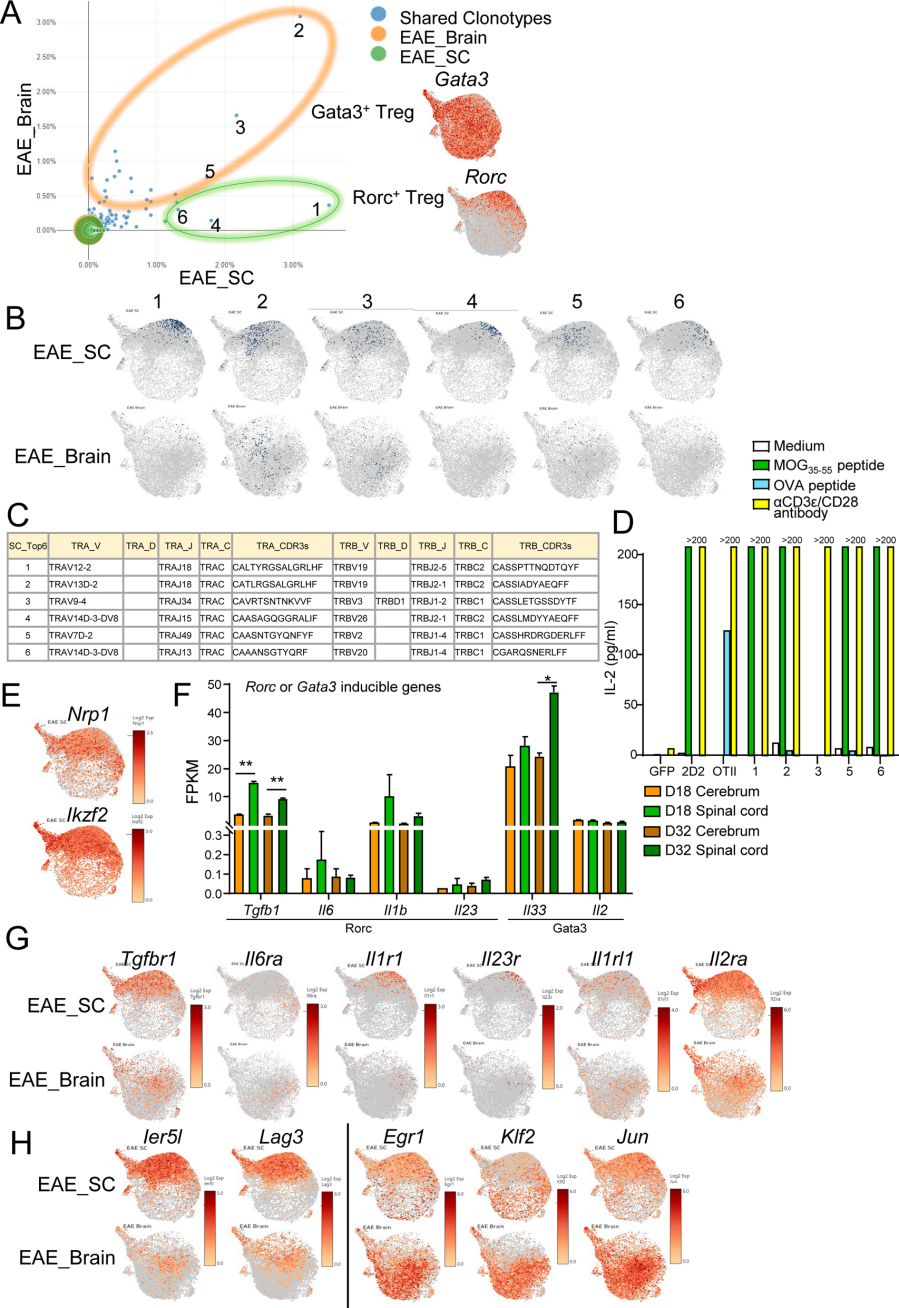
TCR analysis of the brain and spinal cord of EAE mice. **A–C** TCR repertoire analysis of brain and spinal cord Tregs from EAE Foxp3^hCD2KI^ mice (N = 4) at day 23. The plot of the percentage of shared clonotypes between the brain and spinal cord (**A**). Feature plot of the top six TCR clonotypes shared between the brain and spinal cord (**B**). The sequences of the top six TCR clonotypes shared between the brain and spinal cord (**C**). **D** The protein levels of IL-2 in the supernatants of the top six TCR-induced TG40 cells stimulated with MOG_33-55_ peptide (5 μg/ml), OVA peptide (200 μg/ml) or anti-CD3ε/CD28 antibodies (2 μg/ml) for 24 h. **E** Feature plot of tTreg markers such as *Nrp1* and *Ikzf2*. **F** The expression levels of Rorc or Gata3 inducible cytokine genes in bulk RNA-seq data from cerebrum or spinal cord tissues derived from EAE Foxp3^hCD2KI^ mice at 18 or 32 days after immunization with MOG peptide (day 18 and day 32: N = 2, pooled from 3 animals for each replicate). **G** Feature plot of cytokine receptor genes in (**F**). **H** Feature plot of DEGs between brain Gata3^+^ Tregs and SC Gata3^+^ Tregs. For (**D**), data are representative of at least three independent experiments. *P* values were determined by two-tailed Student’s t test (**F**). The data are shown as the mean ± s.e.m

To investigate whether antigen specificity is a critical factor in shaping Treg properties, we performed scTCR-seq analysis of CNS Tregs from EAE mice. The results showed that TCRs with high clonality were present in both the brain and spinal cord (Fig. 4A). We focused on the top six TCR sequences in the spinal cord and examined the cells expressing these TCRs. Rorc^+^ Tregs (Nos. 1, 4, and 6) were detected in the spinal cord but rarely in the brain. In contrast, Gata3^+^ Tregs (Nos. 2, 3, and 5) were frequently detected in both the brain and spinal cord (Fig. 4B, C). Gata3^+^ Tregs are tTregs that recognize self-antigens, whereas Rorc^+^ Tregs are peripheral Tregs (pTregs) derived from naïve T cells influenced by gut bacteria and dietary antigens in the intestinal tract [16–19]. Therefore, we hypothesized that Gata3^+^ and Rorc^+^ Tregs recognize different antigens and cloned TCRs to identify these antigens. We performed in vitro culture experiments expressing the TCR of Treg on TCR-deleted T cell hybridomas producing IL-2 in response to TCR stimulation. Contrary to our expectations, however, both Gata3^+^ Tregs and Rorc^+^ Tregs responded to the myelin oligodendrocyte glycoprotein (MOG) peptide, except for No. 3 (No. 4 was excluded because the cloned TCR did not appear on the T cell surface), suggesting that although CNS antigens are required for CNS infiltration, the observed differences in properties are not antigen dependent (Fig. 4D).

Since it has been suggested that Rorγt-positive Tregs are pTregs [19], we hypothesized that the spinal cord has a tissue environment conducive to Rorc^+^ Tregs and investigated their origin as either tTregs or inducible pTregs. The low expression of *Nrp1* and *Ikzf2* (Helios), which are tTreg markers, in Rorc^+^ Tregs suggested that these cells were likely inducible Tregs (Fig. 4E, S4A, B). The genes involved in the induction of *Rorc* expression (*Tgfb1*, *Il6*, *Il1b*, and *Il23*) tended to be more highly expressed in the spinal cord than in brain tissue, whereas those involved in *Gata3* expression (*Il33* and *Il2*) showed no significant difference between the two tissues (Fig. 4F). Furthermore, the expression of their receptors on Tregs suggested that *Il1r1* and *Il23r* were expressed on Rorc^+^ Tregs (Fig. 4G, S4C). Differences in gene expression between the spinal cord and brain were also detected for Gata3^+^ Tregs; *Ier5l* and *Lag3* were highly expressed in spinal cord Gata3^+^ Tregs; and *Egr1*, *Klf2* and *Jun* were highly expressed in brain Tregs (Fig. 4H, S4C) (some genes are included in Fig. 3C). These Gata3^+^ Tregs have the same TCR but different characteristics depending on the localized tissue, suggesting the importance of the environment, including chemokines and cytokines, rather than antigen specificity.

### Spinal cord Tregs and brain Tregs have different chemokine receptors

**Fig. 5:**
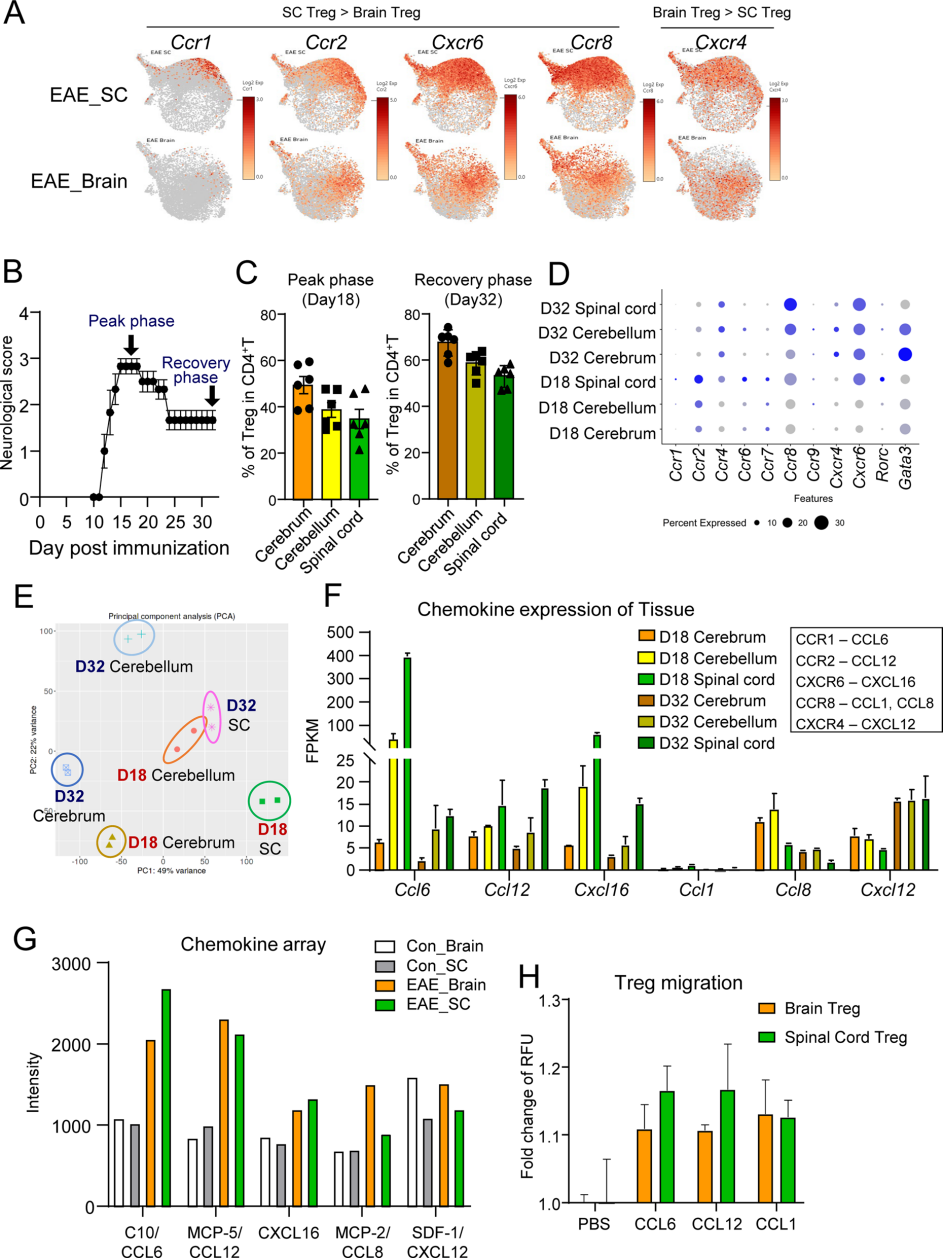
Differences in chemokine-chemokine receptor interactions. **A** scRNA-seq data of Tregs derived from EAE Foxp3^hCD2KI^ mice (N = 4) at day 23. Feature plot of chemokine receptors associated with DEGs between brain Tregs and spinal cord Tregs in Tregs. **B**–**D** Foxp3^hCD2KI^ mice (day 18: N = 6, day 32: N = 6) were subjected to EAE. Cerebrum, cerebellum, and spinal cord Tregs were sorted at 18 and 32 days after immunization. Neurological score of EAE mice (**B**). Flow cytometric analysis of the percentage of Tregs among CD4^+^ T cells in the cerebrum, cerebellum, and spinal cord (**C**). Dot plot of chemokine receptors on Tregs (**D**). **E**, **F** PCA (**E**) and expression levels of chemokines (**F**) determined via bulk RNA-seq analysis of the cerebrum, cerebellum, and spinal cord in mice (N = 2, pooled from 3 animals for each replicate) of **B**–**D**. **G** Chemokine array analysis of brain or spinal cord lysates from untreated or EAE mice at day 23 (N = 3). **H** Migration of brain or spinal cord Tregs derived from EAE Foxp3^hCD2KI^ mice at day 22 in the presence of CCL6, CCL12 or CCL1. For (**H**), data are representative of at least two independent experiments. *P* values were determined by two-tailed Student’s t test (cerebrum vs. spinal cord) (**E**). The data are shown as the mean ± s.e.m

Next, we investigated the relationship between chemokine receptors on Tregs and chemokines in the spinal cord and brain. Bulk RNA-seq, scRNA-seq and flow cytometry analysis revealed that Ccr1, Ccr2, Cxcr6 and Ccr8 were highly expressed as chemokine receptors on spinal cord Tregs, whereas Cxcr4 was highly expressed on brain Tregs (Fig. 5A, S5A-D). To investigate the relationship between Treg localization and chemokine receptors over time, we performed scRNA-seq analysis of Tregs in the cerebrum, cerebellum, and spinal cord during the peak and recovery phases of EAE symptoms (Fig. 5B). From the peak phase onward, the percentage of Tregs in the cerebrum was greater than that in the spinal cord, and it increased throughout the CNS during the recovery phase (Fig. 5C). At the time of high inflammation on day 18 post EAE induction, the expression of chemokine receptors, such as *Ccr1*, *Ccr2*, *Ccr6*, and *Cxcr6,* was high in spinal cord Tregs, and on day 32 during the recovery period, *Ccr8* and *Cxcr4* were highly expressed in the brain and spinal cord (Fig. 5D). In the spinal cord, Rorc^+^ Tregs were observed during inflammation (Fig. 5D).

Tissue Tregs migrate to target tissues after tissue antigens are presented to their lymph nodes [8]. Using scRNA-seq analysis, the expression of *Rorc* or *Gata3* and the clonotype frequencies of TCRs were investigated in the CNS (cerebrum, cerebellum and spinal cord) and in lymph nodes near the spinal cord (superficial cervical, deep cervical, axillary, mediastinal, renal and lumbar) on day 18 or day 32 after EAE induction (Fig. S6A). Common TCRs between the brain, spinal cord and lymph nodes were detected, and *Rorc* or *Gata3* expression was found to be independent of the clonotype of the TCR. On EAE day 18, Rorc-expressing Tregs were found to the similar extent of *Gata3*-expressing cells in populations expressing TCRs, and high clonality was detected in the CNS, especially in the spinal cord (Fig. S6B). On day 32 after EAE, the number of populations expressing *Rorc* was smaller than that of those expressing *Gata3* (Fig. S6C). In both phases, *Rorc* expression was scarcely detected in Tregs in the lymph nodes, except for the cervical lymph nodes. These results suggest that Rorc^+^ Tregs are induced during the acute phase of EAE in the spinal cord rather than in the lymph nodes, independent of the specific antigen.

Next, to examine chemokine expression in the tissues, we performed bulk RNA-seq analysis of the cerebrum, cerebellum, and spinal cord and revealed differences in gene expression in the tissues during the highly inflammatory (day 18) and recovery (day 32) periods (Fig. 5E). The spinal cord exhibited the most inflammation, followed by the cerebellum, while the cerebrum exhibited the least inflammation. Interestingly, the patterns of gene expression in the spinal cord on day 32 and in the cerebellum on day 18 were similar (Fig. 5E). The expression of Ccl6, Ccl12, and Cxcl16, the ligands for Ccr1, Ccr2, and Cxcr6, respectively, was high in the spinal cord on day 18 but decreased during the recovery phase. In contrast, the cerebrum had high expression of Ccl8, the ligand for Ccr8, and Cxcl12. The expression of Cxcl12 increased in the cerebrum, cerebellum, and spinal cord during the recovery period (Fig. 5F, G). Additionally, the cerebellum, which is located between the spinal cord and cerebrum, exhibited intermediate expression (Fig. 5F). According to the results of a chemokine-induced migration assay, spinal cord Tregs tended to migrate more in response to Ccl6 and Ccl12 than brain Tregs. In contrast, Ccl1, the ligand for Ccr8, induced migration of both brain and spinal cord Tregs (Fig. 5H). These results suggest that differences in chemokine expression in the brain and spinal cord may be a determining factor in the characteristics of infiltrating Tregs.

### Tissue-specific Tregs have tissue-specific anti-inflammatory and reparative effects

**Fig. 6:**
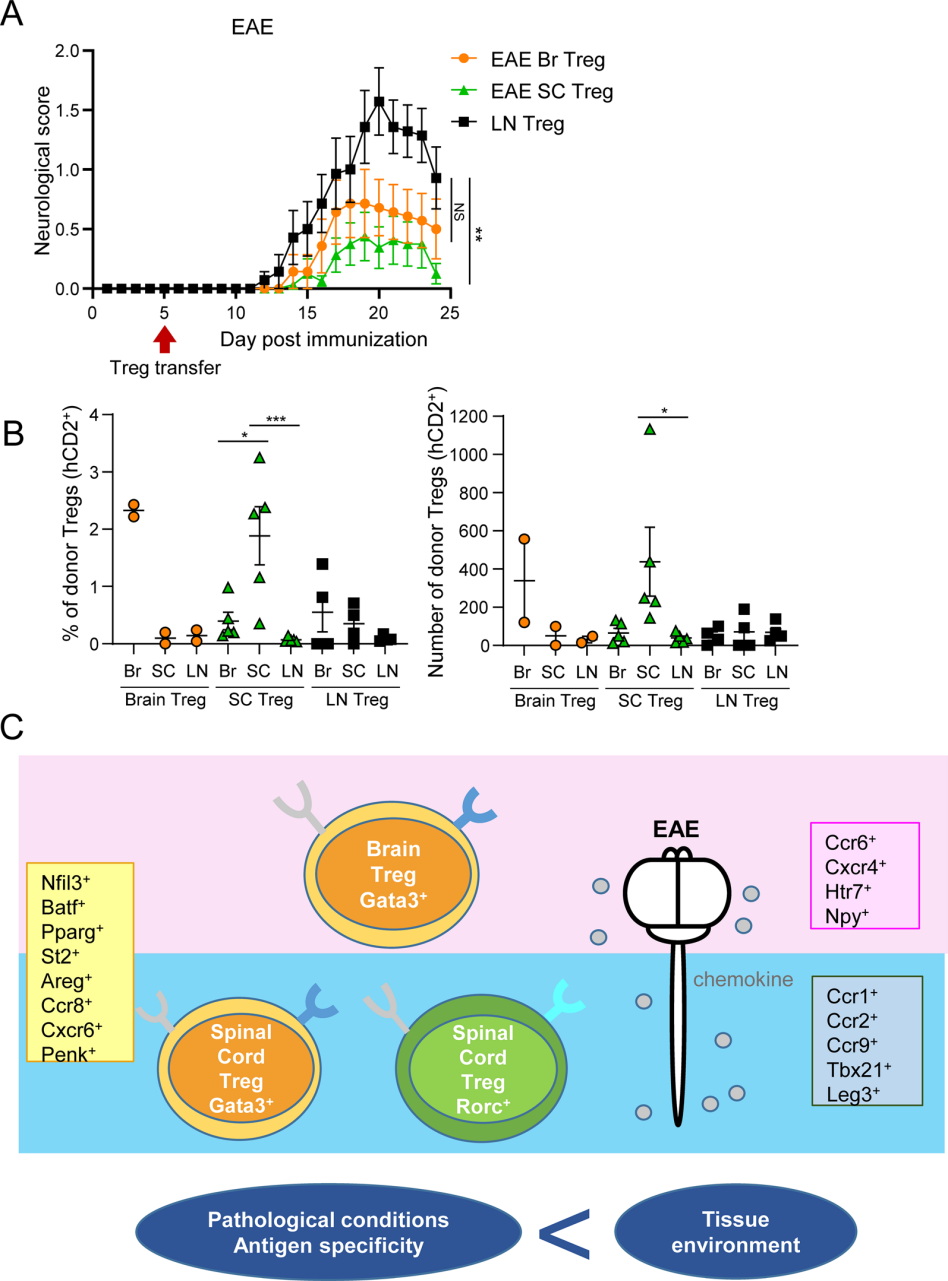
Therapeutic effects of spinal cord Tregs on EAE mice. **A**, **B** Lymph node Tregs from control Foxp3^hCD2KI^ mice, brain Tregs or spinal cord Tregs derived from EAE Foxp3^hCD2KI^ mice at day 23–25 were injected intravenously in EAE wild-type mice at day 5. Neurological scores of EAE wild-type mice (EAE brain Treg; N = 14, EAE spinal cord Treg; N = 16, Lymph node Treg; N = 14). The data were pooled from two independent experiments. *P* values were determined by two-way ANOVA. The data are shown as the mean ± s.e.m (**A**). Flow cytometric analysis of Tregs in the brain, spinal cord or lymph node derived from Tregs-transferred mice (EAE brain Treg; N = 2, EAE spinal cord Treg; N = 5, Lymph node Treg; N = 4). The data are representative of at least two independent experiments. *P* values were determined by one-way ANOVA. The data are shown as the mean ± s.e.m (**B**). **C** Schematic model showing the difference of Treg phenotype between brain and spinal cord. SC: spinal cord, LN: lymph node

To examine the effect of brain and spinal cord Tregs on EAE symptoms, we performed a transfer experiment using Tregs from EAE mice. Tregs from the spinal cord of EAE mice showed a significant alleviation of symptoms compared to Tregs from untreated lymph nodes (Fig. 6A, S7A). In addition, brain Tregs had an ameliorative effect that was intermediate between the effects of spinal cord and lymph node Tregs (Fig. 6A). Analysis of the recipient's brain, spinal cord, and lymph nodes revealed a distinct migratory pattern of Tregs depending on their tissue of origin. Specifically, Tregs originating from the brain were found to preferentially migrate to the brain, whereas those derived from the spinal cord exhibited a higher propensity to migrate to the spinal cord (Fig. 6B). The phenotype of the transferred Tregs was more positive for Helios in Tregs in brain tissue than in Tregs in spinal cord tissue (Fig. S7B). To determine whether Tregs are more important in suppressing disease progression or tissue repair, we transferred Tregs at the peak of EAE symptoms and examined the contribution of Tregs to recovery from EAE. We found that transplantation of Tregs did not contribute to the recovery of neurological scores, but suppressed microglial and astrocyte activation (Fig. S8A, B). These result, combined with the fact that transplantation of Tregs prior to disease onset inhibits disease onset and progression (Fig. 6A), suggests that Treg transplantation may be more effective in reducing inflammation than in tissue repair. These data suggest that spinal cord Tregs are more likely to infiltrate the spinal cord and promote tissue protection in a tissue-specific manner after infiltration.

## Discussion

Although detailed characterization of Tregs in tissues, such as the gut, has been reported, differences in the characteristics of Tregs in the CNS have not been elucidated. In this study, we clarified the differences in Treg phenotypes between the brain and spinal cord using a CNS disease model of ischemic stroke and EAE (Fig. 6C).

The importance of Tregs in CNS diseases has been demonstrated by the exacerbation of the neurological symptoms of ischemic stroke and EAE in the absence of Tregs in DEREG mice or CD25 antibodies [5, 20]. Moreover, Tregs have also shown symptomatic ameliorative effects on other neurological diseases, such as Parkinson’s disease and amyotrophic lateral sclerosis [21–23]. Therefore, it is necessary to understand the different characteristics of Tregs to develop Treg-mediated disease therapies.

Gene expression analysis of brain and spinal cord Tregs from stroke and EAE mice revealed that these cells are more similar to each other than to spleen Tregs but have different properties in each tissue and pathological condition. GO enrichment analysis showed that the genes could be divided into clusters based on their distinct expression in the brain and spinal cord after stroke and in EAE, suggesting that differences in the tissue environment are more important than pathological conditions. Furthermore, analysis of the TCRs of brain and spinal cord Tregs in EAE revealed that highly clonal TCRs are present in both tissues, suggesting that the tissue environment is more important for the acquisition of tissue-specific properties than is the disease state or antigen specificity.

Tissue Tregs can be epigenetically modified by priming with tissue antigens in secondary lymphoid tissues [6–9]. Tregs infiltrating the brain and spinal cord may recognize and clonally proliferate MOG and other autoantigens, but no other self-antigens were identified in this study. Moreover, the presentation of self-antigens may be necessary for Treg survival in the CNS. Thus, given the potential for tissue infiltration by priming with autoantigens in secondary lymphoid tissues, antigen specificity is important in the early stages of the acquisition of tissue-specific phenotypes. The fact that Rorc^+^ and Gata3^+^ Tregs recognize the same MOG peptide for antigens but have different TCR sequences suggests that the TCR is one of the determinants of these properties.

In Gata3^+^ Tregs, GATA3 stabilizes FOXP3 expression, enhances its inhibitory capacity and allows tissue-specific expression of homing receptors, which are involved in suppressing inflammation in the kidney and skin and suppressing fibrosis in Th2 responses [14, 24]. In the intestinal tract, Rorc^+^ Tregs induced from naïve T-cells by gut bacteria and dietary antigens play a major role [16, 18, 19]. Rorc^+^ Tregs are present in the spinal cord in EAE, however, they are rarely present in the brain. This may be because the spinal cord has an environment (e.g., expression of IL-6, IL-1β, IL-23, and TGFβ) that can induce and maintain Rorc^+^ Tregs, whereas the brain may lack such an environment. Another important factor that determines the localization of tissue Tregs is that some chemokines are expressed in the brain and spinal cord, while others are different, and there are also differences in the chemokine receptors on Tregs.

Changes in the types and characteristics of immune cells in the brain and spinal cord occur over time after EAE or stroke. Therefore, the tissue-specific properties of the brain and spinal cord observed in this study may be transient. From the onset to the peak of symptoms, the Th17 response is strong, and chemokines that attract Rorc^+^ Tregs, especially those in the spinal cord, are highly expressed. However, the expression of these chemokines is low in the brain. Chemokine receptors, such as *Cxcr6*, which are highly expressed on almost all CNS Tregs during EAE induction, may be used to infiltrate the spinal cord and are subsequently attracted by chemokines that are highly expressed in the brain. It is possible that after migrating to the brain, their phenotype can change according to the environment. However, the exact routes of infiltration into the brain and spinal cord remain unclear.

We also analyzed the distribution of Tregs in the lymph nodes and the CNS. These results suggested that in the early stage of inflammation, Tregs undergo antigen presentation in the axillary and renal lymph nodes and infiltrate the spinal cord. This may be due to the possibility of antigen presentation in the lymph nodes where the MOG-containing emulsion was injected subcutaneously. Subsequently, Tregs expressing the same TCR as CNS Tregs were detected in the deep cervical lymph nodes, possibly because of their connection with the lymphatic vessels of the dura covering the brain.

The transfer of Tregs isolated from the spinal cord and brain strongly alleviated neurological symptoms in the same tissue as was observed in the isolated tissue, suggesting a tissue-specific suppressive effect. However, it is unclear whether this difference is due to the number of infiltrating Tregs or tissue-specific repair factors. Recent advances in the study of tissue Tregs have revealed that they not only suppress inflammation but also express a variety of characteristic genes, including tissue-specific repair factors and factors that promote stem cell differentiation [4, 25]. The identification of Treg-derived repair factors through a more comprehensive analysis will likely lead to the discovery of potential therapeutic molecules for neuroinflammatory and neurodegenerative diseases.

### Supplementary Information

Additional file 1. Fig. S1 Effect of diphtheria toxin on DEREG mice and the number of tissue Tregs. **A** Flow cytometric analysis of EAE brain and EAE spinal cord Tregs in DEREG mice (N = 3) or wild-type mice (N = 4) at day 17. **B** Flow cytometric analysis of brain or spinal cord Tregs derived from EAE Foxp3^hCD2KI^ mice at day 24 (N = 6) and lung or spleen Tregs from control mice. The data are representative of at least two independent experiments. *P* values were determined by two-tailed Student’s t test. The data are shown as the mean ± s.e.m. SC: spinal cord

Additional file 2. Fig. S2 The analysis of bulk RNA-seq of Treg. **A** Tregs were sorted from ischemia Foxp3^hCD2KI^ mice brain at day 23 (N = 4) or MOG-treated Foxp3^hCD2KI^ mice brain and spinal cord at day 23 (N = 4). Flow cytometry dot-plots show gating strategy for defining Tregs. **B**–**D** Bulk RNA-seq data of brain Tregs or spinal cord Tregs derived from EAE Foxp3^hCD2KI^ mice at day 28 and spleen Tregs from control mice. Gene expression level of Treg markers (**B**). Enrichment analysis of the genes that characterize the PCA eigenvectors in main Fig. 2A (**C**). Gene expression level of marker genes in cluster C and D in Fig. 2C (**D**)

Additional file 3. Fig. S3 The protein expression of transcription factor of Treg. Flow cytometric analysis of brain or spinal cord Tregs derived from EAE Foxp3^hCD2KI^ mice at day 27 (N = 3, pooled from 12 animals for each replicate). *P* values were determined by two-tailed Student’s t test. The data are shown as the mean ± s.e.m. SC: spinal cord

Additional file 4 Fig. S4 The protein expression of tTreg markers and receptors of Tregs. **A**–**C** Flow cytometric analysis of brain or spinal cord Tregs derived from EAE Foxp3^hCD2KI^ mice at day 27 (N = 3, pooled from 12 animals for each replicate). tTreg markers (Helios and Nrp1), RORγt, cytokine receptors (IL1R, ST2 and CD25) and LAG3 were detected. *P* values were determined by two-tailed Student’s t test. The data are shown as the mean ± s.e.m. SC: spinal cord

Additional file 5. Fig. S5 The protein expression of chemokine receptors of Tregs. **A**, **B** Volcano plot showing DEG from bulk RNA-seq data of brain Tregs or spinal cord Tregs derived from EAE Foxp3^hCD2KI^ mice (N = 2, pooled from 4 animals for each replicate) at day 28 (**A**) and scRNA-seq data of Tregs derived from EAE Foxp3^hCD2KI^ mice (N = 4) at day 23 (**B**). **C** The expression level of chemokine receptors in the brains or spinal cords derived from mice shown in (**A**). Spleen Tregs were sorted from control Foxp3^hCD2KI^ mice. The data are shown as the mean ± s.e.m. **D** Flow cytometric analysis of brain or spinal cord Tregs derived from EAE Foxp3^hCD2KI^ mice at day 24 (N = 3, pooled from 2 animals). Right bar graphs were obtained by left representative histograms. *P* values were determined by two-tailed Student’s t test. The data are shown as the mean ± s.e.m. SP: spleen, SC: spinal cord

Additional file 6. Fig. S6 Rorc or Gata3 expression pattern in Tregs expressing CNS or lymph node TCRs in EAE mice. **A** Schematic representation of the investigated lymph nodes. **B**, **C** Analysis of the TCR repertoire and gene expression using scRNA-seq at EAE day 18 (**B**) or day 32 (**C**) after immunization. The left images show the clonotype frequency of the TCRs. The right images represent tissue-specific *Rorc* and *Gata3* expression patterns according to TCR clonotype

Additional file 7. Fig. S7 Tregs sorting by flow cytometry. **A** Tregs were sorted from MOG-treated Foxp3^hCD2KI^ mice brain or spinal cord at EAE day 23 (N = 34) or CFA-treated Foxp3^hCD2KI^ mice spleen (N = 4). Flow cytometry dot-plots show gating strategy for defining Tregs. **B** Flow cytometric analysis of transferred brain or SC Tregs. Brain Tregs and SC Tregs sorted from EAE Foxp3^hCD2KI^ mice at day 28 were injected intravenously in EAE wild-type mice at day 5. Brain cells in brain Treg-injected mice and spinal cord cells in spinal cord Treg-injected mice were analyzed by flow cytometry. *P* values were determined by two-tailed Student’s t test. The data are shown as the mean ± s.e.m. SP: spleen, SC: spinal cord

Additional file 8. Fig. S8 The effects of spinal cord Tregs on EAE mice. **A**, **B** Tregs derived from spleen of CFA-treated Foxp3^hCD2KI^ mice (Spleen Treg) or brain or spinal cord of MOG-treated Foxp3^hCD2KI^ mice (Brain Treg and SC Treg, respectively) or only PBS were injected intravenously at day 17 in wild-type mice that were subjected to EAE. (PBS; N = 6, Spleen Treg; N = 6, Brain Treg; N = 7, SC Treg; N = 6.) Clinical score of recipient mice (**A**). Spinal cords of EAE mice at day 27 derived from (**A**) were stained with luxol fast blue (LFB) & crecyl violet (CV), antibodies targeting MBP, Iba1 or GFAP. Representative images were shown. Images were obtained by 20 objective. Scale bar; 100 µm. To assess the level of gliosis or accumulation of myeloid cells, Iba1 or GFAP positive area was measured (**B**). *P* values were determined by one-way ANOVA. The data are shown as the mean ± s.e.m.

## Data Availability

Bulk RNA-seq data for Tregs are available under accession number GSE252867. Bulk RNA-seq data from the brain and spinal cord are available under accession number GSE252641. scRNA-seq data for Tregs are available under accession number GSE252368.
